# Pressure Pain Threshold Cut-Off Points at Trigeminal and Extra-Trigeminal Nervous and Musculoskeletal Structures to Discriminate Patients with Migraine from Episodic Tension-Type Headache: A Diagnostic Accuracy Study

**DOI:** 10.3390/diagnostics16060823

**Published:** 2026-03-10

**Authors:** Leandro H. Caamaño-Barrios, Naiara Benítez-Aramburu, Alberto Nava-Varas, Fernando Galán-del-Río, Mónica López-Redondo, Jorge Buffet-García, Ricardo Ortega-Santiago

**Affiliations:** 1 Escuela Universitaria Gimbernat Cantabria, Adscrita la Universidad de Cantabria, 39300 Torrelavega, Spain; leandro.caamano@eug.es (L.H.C.-B.); naiara.benitez@eug.es (N.B.-A.); 2 Escuela Internacional de Doctorado, Universidad Rey Juan Carlos, 28922 Alcorcón, Spain; albertonavavaras@gmail.com; 3 Department of Physical Therapy, Occupational Therapy, Rehabilitation and Physical Medicine, Universidad Rey Juan Carlos, 28922 Alcorcón, Spain; fernando.galandel@urjc.es; 4 Grupo de Investigación de Alto Rendimiento en Evaluación Multidimensional y Tratamiento del Dolor Crónico, Universidad Rey Juan Carlos, 28922 Alcorcón, Spain; 5 Faculty of Health Sciences, Francisco de Vitoria University, 28223 Madrid, Spain; monica.lopezredondo@ufv.es

**Keywords:** diagnostic accuracy, mechanical hyperalgesia, migraine, pressure pain threshold, tension-type headache

## Abstract

**Background/Objectives**: Pressure pain thresholds (PPTs) are commonly used to quantify mechanical hyperalgesia in migraine and tension-type headache (TTH), but the discriminatory performance of PPTs across neural and muscular sites remains unclear. This study compared nerve- and muscle-related PPTs between migraine and frequent episodic TTH and explored site-specific ROC-derived cut-off values as complementary classification markers. **Methods:** In this cross-sectional case-group discrimination study, participants with migraine (*n* = 33) and frequent episodic TTH (*n* = 31) underwent bilateral PPT assessment (electronic algometry) over the temporalis and tibialis anterior muscles, C5/C6 zygapophyseal joints, peripheral nerves (greater occipital, median, ulnar, radial, posterior tibial, common peroneal), and the second metacarpal region. **Results:** PPTs were generally lower in the migraine group than in the TTH group. After adjustment for sex and age, the most consistent between-group differences remained at the temporalis muscles bilaterally (left: adjusted mean difference 0.49 kg/cm^2^, 95% CI 0.10 to 0.89, *p* = 0.015; right: 0.53 kg/cm^2^, 95% CI 0.13 to 0.93, *p* = 0.011) and at the left tibialis anterior muscle (0.90 kg/cm^2^, 95% CI 0.03 to 1.78, *p* = 0.044). In the main ROC analysis, the temporalis muscles showed the strongest discriminatory performance (left AUC = 0.733; right AUC = 0.707), whereas tibialis anterior and left posterior tibial nerve sites showed modest, below-threshold discrimination (AUCs < 0.70 despite statistical significance in some cases). Women-only ROC analyses showed a broadly similar pattern, with slightly improved metrics at some sites, particularly the temporalis muscles. Across most sites, likelihood ratios indicated only small-to-moderate shifts in post-test probability. **Conclusions:** Participants with migraine showed lower PPTs than those with frequent episodic TTH across most assessed sites, with the clearest differences at the temporalis muscles. ROC and PR analyses suggest that PPTs (especially at temporalis sites) may provide complementary, hypothesis-generating discriminatory information, but their overall stand-alone discriminative utility is modest. PPT assessment should therefore be interpreted as an adjunct to clinical evaluation rather than a replacement diagnostic test.

## 1. Introduction

The International Headache Society describes headache as any painful experience felt in the head, which can also radiate toward the neck. According to the International Classification of Headache Disorders (ICHD-3), headaches are organized into primary and secondary forms based on clinical features and accompanying symptoms [1].

Migraine typically consists of recurrent headache attacks with a throbbing or pulsatile quality. These episodes vary in frequency, severity, and duration, are often unilateral, tend to worsen with physical exertion, and may be accompanied by nausea and vomiting [2]. A subset of patients also experience transient, fully reversible neurological symptoms known as migraine aura [1,3]. In contrast, tension-type headache (TTH) commonly presents as bilateral pain with a pressing or tightening sensation, usually of mild to moderate intensity, lasting from minutes to several days [4]. Symptoms generally do not intensify with physical activity and are not associated with nausea, although photophobia and/or phonophobia can occur. Cervicogenic headache, by comparison, is a secondary headache attributed to pathology in the cervical spine and related structures, including bony, myofascial, and/or disc components [5]. It is typically unilateral, may be intermittent or continuous, and is frequently linked to restricted cervical range of motion. Pain and associated symptoms are often triggered by specific neck movements, sustained postures, or palpation/pressure applied to posterior cervical regions [3,6,7].

Epidemiological data consistently indicate that primary headache disorders are the most common. TTH is considered the most prevalent headache condition worldwide, affecting roughly 26–27% of the global population [8]. An estimated 2 billion people experienced TTH in 2021 [9], and global prevalence increased by around 37% between 1990 and 2019 [10,11]. Although less frequent than TTH, migraine remains a substantial source of worldwide disability, with approximately 1 billion affected individuals in 2021 [12], and a marked sex difference, as women are affected about twice as often as men (20.7% vs. 9.7%) [13].

In both migraines and TTH, altered nociceptive processing is commonly expressed as hyperalgesia to experimentally evoked stimuli assessed with quantitative sensory testing (QST) [14]. In headache research, QST most commonly uses mechanical pressure, thermal, and electrical stimuli to assess pain responsiveness [15]. Among these methods, pressure algometry is especially feasible in clinical settings, as it captures mechanosensitivity by measuring pressure pain thresholds (PPTs) [16].

Nahman-Averbuch et al. [14] reported that people with migraine exhibited lower pain thresholds to heat and pressure than headache-free controls. For pressure algometry, reduced PPTs were consistently observed when testing cranio-cervical regions, whereas this pattern was not reliably reproduced at extra-trigeminal sites, indicating that the anatomical territory examined strongly shapes the results. The same review also reported increased pain responses to suprathreshold stimulation in some conditions, including higher pain ratings to cold and electrical stimuli depending on the region assessed. These findings support the view that QST abnormalities in migraine are dependent on the stimulus modality, the outcome measure, and the testing site, and they reinforce the clinical relevance of PPTs as a feasible marker of mechanosensitivity in this population.

In addition, the importance of assessing PPTs is also supported by previous network-based analyses of patients with TTH [17]. PPT measures emerged as closely connected psychophysical indicators. Thresholds obtained at cranio-cervical and remote body regions tended to group within the same network and showed strong positive intercorrelations, including a clear linkage between cervical spine and temporalis sites. These PPT patterns were also directly related to sex (with greater pressure sensitivity in women) as well as to depressive symptoms and reduced vitality.

Recent diagnostic-accuracy research has investigated whether PPT-derived thresholds can discriminate headache-free individuals from those with migraine or TTH, and whether they can separate frequent episodic from chronic forms of both disorders [18,19]. However, these studies assessed PPTs at a limited number of locations (temporalis, C5-C6, second metacarpal, and tibialis anterior) and were largely restricted to muscular or segmental landmarks, without incorporating measurements along peripheral nerve trajectories or projection areas. Consequently, further work is needed. Broadening PPT mapping to encompass both muscle sites and nerve-related regions could overcome these limitations and clarify which testing locations provide the best balance of sensitivity and specificity for diagnostic discrimination.

Accordingly, the aims of the present study were to compare nerve- and muscle-related tissue sensitivity across cranial, cervical, and distal regions between individuals with migraine and frequent episodic TTH and to explore the discriminatory performance of PPT-derived cut-offs for distinguishing migraine from TTH, defined by the best balance of sensitivity, specificity, and positive and negative likelihood ratios (LRs).

## 2. Materials and Methods

### 2.1. Study Design

This was a cross-sectional case-group study designed to evaluate the discriminative performance of PPT measurements at various anatomical locations for differentiating participants with migraine from those with episodic TTH. In the present case-group discrimination design, the index test was PPT assessment and the reference standard was the clinical diagnosis of migraine or frequent episodic TTH established with a structured clinical assessment performed by the neurologist conducted by an experienced neurologist according to ICHD-3-based criteria and study eligibility criteria. Therefore, the study was designed to evaluate ROC-based discriminatory/classification performance of PPTs between clinically diagnosed groups, rather than diagnostic replacement testing in a setting of diagnostic uncertainty.

To ensure methodological rigor, the study followed the Updated List of Essential Items for Reporting Diagnostic Accuracy Studies (STARD) [20] and the Enhancing the QUAlity and Transparency Of health Research (EQUATOR) guidelines [21]. The study protocol, including ethical considerations and protection of participants’ rights, was reviewed and approved by the Cantabria Research and Drugs Ethics Committee prior to the initiation of data collection (2016/104) on 16 December 2016.

### 2.2. Participants

Consecutive women diagnosed with migraine and episodic TTH by an experienced neurologist (reference standard) were enrolled at a university-based center in Cantabria (Spain) between January 2024 and May 2025. Migraine diagnosis followed the third edition of the International Classification of Headache Disorders [1]. Informed consent was obtained from all subjects involved in the study.

#### 2.2.1. Eligibility Criteria for Patients with Migraine

A comprehensive migraine diary profile was obtained, covering pain distribution and characteristics, years since onset, attack frequency and severity, family history of migraine, and current medication intake. Participants were not eligible if they had: (1) any additional primary or secondary headache disorder, including medication-overuse headache; (2) prior head or neck injury (e.g., whiplash); (3) pregnancy; (4) cervical disc herniation; (5) relevant medical comorbidities (e.g., rheumatoid arthritis); (6) other chronic pain conditions (e.g., fibromyalgia or cancer-related pain); or (7) undergone any medical procedure within the last three months, including aesthetic block interventions. In addition, participants had to be assessed interictally (i.e., outside a migraine attack), and in case of females, when they were not menstruating at the time of testing.

#### 2.2.2. Eligibility Criteria for Patients with Frequent Episodic TTH

For the episodic TTH group, inclusion required bilateral headache, 15–179 headache days per year [22], mild-to-moderate pain intensity (3–6 on a 0–10 numerical pain rating scale) [23], and a pressing/tightening pain quality. Headache symptoms could not worsen with physical activity and had to occur in the absence of nausea and/or vomiting, although photophobia or phonophobia could be present. Only women with frequent episodic TTH were enrolled. Exclusion criteria comprised chronic TTH, any other primary or secondary headache, fibromyalgia, cervical disc herniation or cervical osteoarthritis based on clinical history, previous cranial or cervical trauma, systemic degenerative disease, having received anesthetic blocks or any physical therapy within the previous six months, or pregnancy.

#### 2.2.3. Sample Size Calculation

Sample size calculation was determined using the G*Power software (v.3.1. for Mac OS) based on detecting a moderate-to-large effect size (0.75) between the tension-type headache and migraine groups, using a two-tailed test, an alpha level of 0.05, and a statistical power (β) of 90%. This resulted in a required minimum of 30 participants per group.

### 2.3. Pressure Pain Sensitivity

Pressure pain thresholds (index test) were measured using an electronic algometer with a 1 cm^2^ round probe tip (Somedic AB, Farsta, Sweden), and results were expressed in kg/cm^2^/s. Pressure was applied with an approximate ramp of 0.3 kg/cm^2^/s, and participants were instructed to activate a handheld switch at the moment pressure first became painful. Three assessments were collected at each location, allowing 30 s of rest between trials, and the mean of the three recordings was used for subsequent analyses. This procedure has demonstrated high reliability [24].

Prior to the assessment session, participants attended a brief familiarization visit to ensure consistent understanding of the protocol. Women with frequent episodic TTH were tested on a headache-free day and were instructed to avoid analgesic intake during the 24 h preceding the evaluation. All PPT measurements were obtained bilaterally by a physiotherapist blinded to group allocation. Assessments were conducted over the following peripheral nerves: greater occipital, median, ulnar, radial, posterior tibial, and common peroneal. Additional sites included the temporalis and tibialis anterior muscles and the C5/C6 zygapophyseal joints. For analytical purposes, locations were categorized as trigeminal/symptomatic (greater occipital nerve and temporalis), extra-trigeminal (median nerve at C5, radial nerve at C6, ulnar nerve at C7, second metacarpal region, and the C5/C6 joint), and a distal pain-free territory (common peroneal nerve at S1, posterior tibial nerve at L1, and tibialis anterior). This classification was used as an interpretive framework for potential regional versus more widespread mechanical hypersensitivity, rather than as a formal neuroanatomical boundary definition.

Anatomical landmarks were identified by manual palpation and marked with a skin pencil before testing. Previous work indicates that PPT assessment over nerve tissue shows moderate-to-high reliability [25]. Measurements were obtained bilaterally at all sites to characterize side-specific patterns and avoid masking potential asymmetries.

Minimal detectable change (MDC) values for PPTs have not yet been established specifically for headache populations. In individuals with acute neck pain, MDC estimates of 47.2 kPa for cervical measurements and 98 kPa for the tibialis anterior have been reported [26]. In addition, Ylinen et al. [27] proposed that between-group differences greater than 20% may be interpreted as clinically meaningful.

### 2.4. Statistical Analysis

Statistical analyses were performed using IBM SPSS Statistics v29 for macOS (IBM Corp., Armonk, NY, USA). All tests were two-tailed, with statistical significance set at *p* < 0.05.

Descriptive statistics were then computed separately for the TTH and migraine groups. Given the imbalance in sex distribution between groups and the observed between-group age differences, between-group comparisons in PPTs were analyzed using general linear models, with group as the fixed factor and sex and age included as covariates. Adjusted mean differences, 95% confidence intervals (95% CI), *p* values and effect sizes for the group effect (expressed as partial eta squared, η*p*^2^ were reported).

The discriminative performance of PPTs at each anatomical site for distinguishing TTH from migraine participants was examined using receiver operating characteristic (ROC) curve analysis and the area under the curve (AUC). AUC values ≥ 0.70 were interpreted as indicating acceptable discrimination [28]. For each site, the optimal PPT threshold was identified using the Youden index, and associated diagnostic indices were calculated, including sensitivity, specificity, and positive and negative likelihood ratios (LR+ and LR−). Diagnostic performance was considered acceptable when sensitivity was ≥70% and specificity was ≥50% [29]. To complement ROC-based discrimination analyses, precision-recall (PR) curves were also inspected. PR curves represent the relationship between precision (positive predictive value) and recall (sensitivity) over the full range of thresholds and provide additional information on classification behavior, particularly regarding the proportion of predicted positives that are true positives. In this study, PR curves were used to qualitatively compare the relative performance of PPT sites and to support interpretation of ROC findings, likelihood ratios, and cut-off behavior. PR analyses were considered exploratory.

## 3. Results

No indeterminate PPT measurements were recorded, and no missing data were present for the variables included in the main analyses. Therefore, complete-case analyses were performed including a total of 64 participants, divided equally into a TTH group (*n* = 31; 100% females) and a migraine group (*n* = 33; 67.7% females). Age differences were observed between the groups (migraine group 21.5 ± 1.4 years; TTH group 19.6 ± 4.3 years; *p* = 0.032). Intra-examiner of the three repeated PPT measurements showed good-to-excellent intra-examiner consistency, with values ranging from 0.742 to 0.928 (across assessed sites).

As shown in Table 1, PPTs were consistently lower in the migraine group than in the TTH group, indicating greater mechanical hyperalgesia among participants with migraine. After sex and adjustments, the most consistent between-group differences remained at the temporalis muscles bilaterally (left: adjusted mean difference 0.49 kg/cm^2^, 95% CI 0.10 to 0.89, *p* = 0.015; right: 0.53 kg/cm^2^, 95% CI 0.13 to 0.93, *p* = 0.011), with small-to-moderate effect sizes (partial η*p*^2^ = 0.094 and 0.105, respectively). At extracephalic sites, the left tibialis anterior muscle also remained significantly different after adjustment (adjusted mean difference 0.90 kg/cm^2^, 95% CI 0.03 to 1.78, *p* = 0.044; partial η*p*^2^ = 0.065). By contrast, several differences that were statistically significant in the unadjusted analysis were attenuated after covariate adjustment and no longer reached statistical significance, including the left tibialis posterior nerve (adjusted mean difference 0.48 kg/cm^2^, 95% CI −0.02 to 0.98, *p* = 0.060), right tibialis anterior muscle (0.72 kg/cm^2^, 95% CI −0.03 to 1.47, *p* = 0.061), right 2nd–3rd interdigital hand space (0.37 kg/cm^2^, 95% CI −0.13 to 0.87, *p* = 0.139), and right Arnold nerve (0.18 kg/cm^2^, 95% CI −0.21 to 0.57, *p* = 0.367). Overall, the clearest between-group differences were concentrated at the temporalis muscles and, to a lesser extent, the left tibialis anterior muscle, whereas most upper-limb nerve sites did not show significant differences.

**Figure 1:**
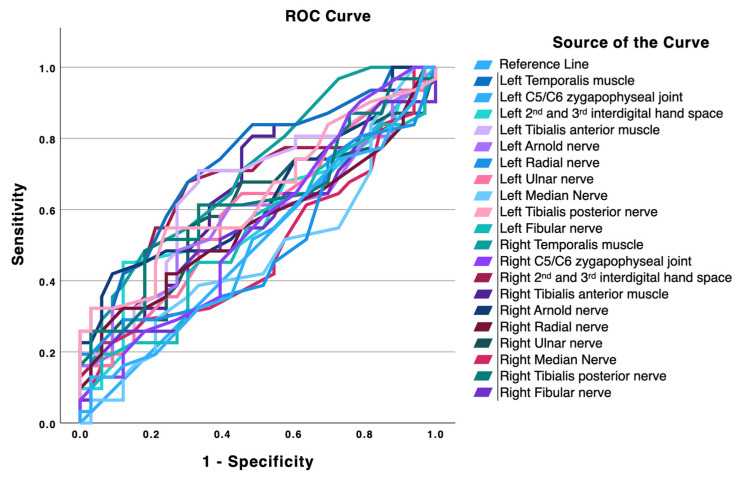
Receiver operating characteristic (ROC) curves for pressure pain thresholds measured over different anatomical sites to discriminate patients with migraine from tension-type headache. Each colored line represents one anatomical location; the diagonal line indicates no discriminative capacity.

Table 2 and the accompanying ROC plot (Figure 1) show that the temporalis muscles were the best single-site discriminators for distinguishing migraine from TTH, with both sides reaching the predefined threshold for acceptable discrimination (AUC ≥ 0.70). The left temporalis achieved an AUC of 0.733 (95% CI 0.609–0.856; *p* < 0.001) with an optimal cut-off of 1.65 kg/cm^2^, yielding sensitivity 0.677, specificity 0.697, Youden index 0.374, positive likelihood ratio (LR+) 2.23 and negative likelihood ratio (LR−) 0.46. The right temporalis reached an AUC of 0.707 (*p* = 0.001) at a 1.95 kg/cm^2^ cut-off (sensitivity 0.484, specificity 0.818, Youden 0.302, LR+ 2.66, LR− 0.63). The tibialis anterior muscles and the left posterior tibial nerve showed modest discriminatory performance and did not reach the predefined threshold for acceptable discrimination, despite statistically significant ROC curves in some cases (left tibialis anterior AUC 0.663, *p* = 0.018; right tibialis anterior AUC 0.665, *p* = 0.016; left posterior tibial nerve AUC 0.641, *p* = 0.045). Among these, the left tibialis anterior showed the most balanced sensitivity and specificity (0.710 and 0.667, respectively; Youden 0.376; LR+ 2.13; LR− 0.44), whereas the right tibialis anterior prioritized sensitivity (0.806) at the expense of specificity (0.515). Most remaining sites clustered around chance-to-low discrimination levels (AUC ≈ 0.50–0.59) and were not statistically different from the diagonal. Notably, some apparently high LR+ values stem from very low false-positive rates at the selected cut-offs despite poor overall AUCs (for example, the left C5-C6 zygapophyseal pillar [AUC 0.496, *p* = 0.952] and the left radial nerve [AUC 0.501, *p* = 0.990, LR+ undefined due to 1-specificity = 0]) and should not be over-interpreted.

**Figure 2:**
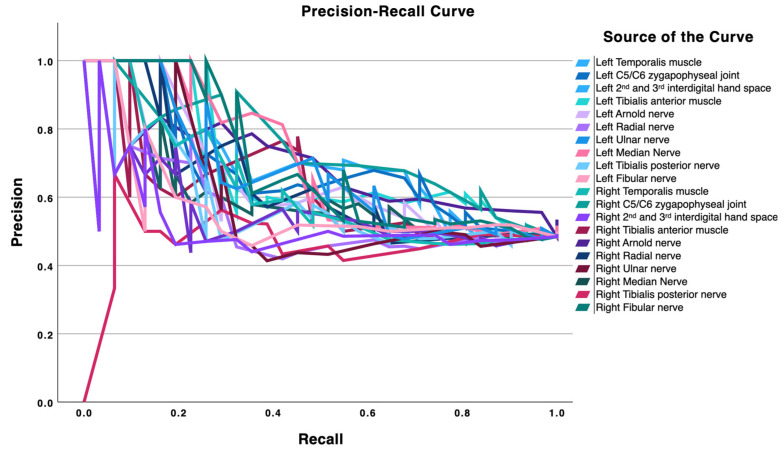
Precision-recall curves for pressure pain thresholds, illustrating the relationship between precision (positive predictive value) and recall (sensitivity) for discriminating patients with migraine from episodic tension-type headache controls at each anatomical site.

The PR plot (Figure 2) complements these findings. As recall increases, precision declines toward intermediate values for most locations, with the temporalis and tibialis anterior traces maintaining comparatively higher precision across moderate recall ranges. This pattern indicates that PPTs at these sites are useful as supportive markers (capable of meaningfully shifting post-test probability with LR+ around 2–3 and LR− near 0.4–0.6) yet are unlikely to function as stand-alone diagnostic tests. Overall, craniofacial PPTs, particularly at the temporalis muscle, provide the most informative discrimination, and lower-limb extracephalic sites add incremental value, in line with the group differences observed in Table 1 and the trajectories depicted in both figures.

Additionally, the results from a specific ROC analysis restricted to women from both groups are reported in Table 3. Overall, the women-only analysis showed a pattern broadly consistent with the main ROC analysis (Table 2), with the temporalis muscles remaining the strongest single-site discriminators and tibialis anterior (specially the left side) and the left posterior tibial nerve showing modest discriminatory ability. However, restricting the analysis to women generally improved discrimination metrics at several sites, especially for the temporalis muscles and some peripheral locations.

Compared with the full ROC analysis, the left temporalis muscle showed improved performance in the women-only analysis while maintaining the same cut-off and sensitivity but with improved specificity. A similar improvement was observed for the right temporalis muscle, again with the same cut-off and sensitivity but lower false-positive rate. At extracephalic sites, the left tibialis anterior muscle also improved in the women-only analysis with the same cut-off. The left posterior tibial nerve remained statistically significant in both analyses, with a small increase in AUC but slightly lower Youden index and LR+ in the women-only analysis. On the right side, the 2nd−3rd interdigital hand space improved modestly and remained significant.

Some variables changed more noticeably in terms of the selected cut-off and/or diagnostic profile (e.g., left C5/C6 zygapophyseal joint, left ulnar nerve, left median nerve, left fibular nerve, right radial nerve, and right tibialis anterior muscle), but most of these sites remained in the low-discrimination range and were not statistically significant. As in the main analysis, several apparently high LR+ values in Table 3 occurred at sites with poor overall AUCs and non-significant ROC curves, likely reflecting low false-positive rates at the chosen cut-offs, and therefore should be interpreted cautiously.

## 4. Discussion

The results of this study demonstrate a generalized reduction in PPTs in women with migraine compared with women with TTH. Lower PPT values were observed across all assessed locations in the migraine group, with the largest differences found bilaterally in muscular tissues (i.e., in the temporalis and tibialis anterior muscles) and unilaterally in the right interdigital 2–3 space. Regarding neural structures, the greatest differences were identified bilaterally in the posterior tibial nerves and unilaterally in the right greater occipital nerve (GON). ROC curve analyses indicated that, among the evaluated sites, the temporalis muscles showed the strongest discriminatory performance, while the tibialis anterior muscles, the right interdigital 2–3 space, and the left posterior tibial nerve showed comparatively better (but generally modest) discriminatory performance. PR plots revealed a consistent pattern for the temporalis and tibialis anterior regions, suggesting their potential utility as supportive diagnostic markers.

### 4.1. Differences in Neural Tissue Sensitivity

Most available evidence on PPTs has traditionally focused on muscular tissues, with far fewer studies examining neural tissues. Among the neural structures assessed in the present study, the right greater occipital nerve showed the most pronounced difference between women with migraine and those with TTH, indicating greater trigeminal sensitivity in migraine. This finding is novel, as Bovim et al. [30] previously reported no significant PPT differences between migraine and TTH groups. The present results may suggest greater involvement or impairment of the GON in migraine pathophysiology. Its known convergence with trigeminal pathways (particularly via the trigeminocervical complex and connections with the supraorbital branch of the trigeminal nerve) may partially account for headache expression in migraine [31,32].

Another noteworthy finding was the bilateral reduction in PPTs in the posterior tibial nerves, with significant between-group differences. These results indicate increased distal and pain-free region hypersensitivity in women with migraine relative to women with TTH. Previous research by our group has shown that women with TTH present neural tissue sensitivity changes compared with healthy controls [33], the present data suggest an even greater degree of sensitization in women with migraine. Although no statistically significant differences were found in other neural sites, it is important to highlight that PPT values were consistently lower in the migraine group across all neural assessments, reinforcing the pattern of increased neural sensitivity.

Regarding ROC curve analyses, the left posterior tibial nerve was the only neural structure demonstrating a discrimination capacity slightly below the acceptable threshold. No other neural structures showed significant between-group discrimination. Although the right GON yielded borderline values, these results should be interpreted cautiously, as the AUC did not meet the ≥0.70 benchmark. Given the lack of prior studies exploring ROC-based discriminative capacity of neural PPTs in primary headaches, future research should address this gap, recognizing the nerve as a mechanosensitive structure capable of generating, expressing, and being affected by sensitization.

Beyond trigeminocervical convergence mechanisms, the present findings may also be interpreted within broader models of pain sensitization and modulation [34]. Reduced PPTs at cephalic sites (e.g., temporalis and greater occipital region) may be compatible with peripheral sensitization and enhanced trigeminal/trigeminocervical nociceptive processing, whereas reduced PPTs at extra-trigeminal and distal pain-free sites are more consistent with widespread hyperalgesia and may reflect central sensitization-related mechanisms and/or altered pain modulation [35]. In this context, the observed PPT pattern may be influenced not only by regional nociceptive sensitization but also by generalized changes in nociceptive gain, including potential impairment of descending inhibitory control. However, because this study assessed mechanical pain sensitivity using PPTs rather than direct neurophysiological markers of sensitization or pain modulation, these mechanistic interpretations should be considered indirect and hypothesis-generating. The present data support a phenotypic pattern of widespread mechanical hypersensitivity, but they do not allow direct differentiation between peripheral sensitization, central sensitization, and altered pain modulation mechanisms.

### 4.2. Differences in Muscular Tissue

Lower PPTs were identified across all muscular locations in the migraine group compared with the TTH group. The most clinically relevant findings were the marked bilateral reductions in PPTs over the temporalis muscles, with greater differences on the left side than the right. These results suggest greater trigeminal involvement in migraine, consistent with neuroinflammatory mechanisms and subsequent signaling within the Gasserian ganglion, a structure known to display heightened inflammatory activity [36,37]. Additional locations demonstrating heightened sensitivity in the migraine group included the right interdigital 2–3 space and both tibialis anterior muscles. Although prior studies have documented PPT reductions in both migraine and TTH [18,38,39], the current findings suggest more pronounced generalized sensitization in women with migraine. This aligns with the increased pressure-pain sensitivity (IPS) phenotype described by Castaldo et al., particularly concerning extra-trigeminal and distal tissue changes [40]. Overall, all PPTs (across both neural and muscular tissues) were lower in the migraine group, supporting a consistent pattern of generalized sensitization.

Regarding ROC analysis, discrimination between migraine and TTH was modest, although both temporalis muscles demonstrated acceptable AUC values. Combined with their sensitivity and specificity, these findings identify the temporalis muscles as clinically meaningful discriminative sites. ROC analysis established reference cut-off points of 1.65 kg/cm^2^ and 1.95 kg/cm^2^ for the left and right temporalis muscles, respectively.

Both tibialis anterior muscles showed modest discriminatory performance. Although their ROC curves were statistically significant, their AUC values (left 0.663; right 0.665) remained below the predefined acceptable threshold (AUC ≥ 0.70). The left tibialis anterior showed a relatively balanced sensitivity/specificity profile, whereas the right side prioritized sensitivity at the expense of specificity. Similarly, the right interdigital 2–3 space and the left posterior tibial nerve showed below-threshold but potentially informative discriminatory performance. Overall, aside from the temporalis muscles, most sites clustered in the low-to-modest discrimination range (approximately AUC 0.50–0.66).

Despite the higher AUC values for the temporalis muscles, the corresponding likelihood ratios indicate only limited diagnostic shifts when interpreted in isolation. In particular, LR+ values in the range of approximately 2–3 and LR− values around 0.4–0.6 are compatible with small-to-moderate changes in post-test probability rather than strong confirmatory or exclusionary performance. Although some sites displayed LR+ > 4 (e.g., left C5-C6 zygapophyseal joint and right GON), these values should be interpreted cautiously because they occurred in the context of low AUCs, non-significant or borderline ROC curves, and/or poor sensitivity, suggesting instability driven by low false-positive rates rather than robust discrimination.

Further research is warranted to enhance diagnostic utility in the regions assessed, as recent pediatric TTH studies report limited discriminative capacity of PPTs in similar anatomical locations, and findings in women with migraine mirror these limitations when distinguishing chronic from episodic migraine [37,41].

Despite the higher AUC values for the temporalis muscles, LRs indicate limited diagnostic confirmatory value. Although some sites displayed LR+ > 4 (e.g., left C5-C6 zygapophyseal joint and right GON), these results require careful interpretation due to low AUC values and poor sensitivity.

PR plots for the temporalis and tibialis anterior muscles support their potential utility as supportive/complementary markers, particularly because they maintained comparatively higher precision across moderate recall ranges, despite only modest likelihood ratios and below-threshold AUCs at the tibialis anterior sites.

### 4.3. Limitations

Certain limitations should be acknowledged. First, the TTH group included only patients with frequent episodic TTH. Therefore, since the findings cannot be generalized to chronic TTH, further analysis for episodic and chronic forms with subgroup differentiation are needed. Second, there was an imbalance in sex distribution between groups (the TTH group included only women, whereas the migraine group included both women and men), and there were also between-group differences in age. Given the known influence of sex and age on pain sensitivity, these factors may have affected PPT comparisons and ROC-derived diagnostic estimates. To mitigate this issue, adjusted analyses including sex and age as covariates were performed, and a women-only sensitivity analysis was conducted. However, because no men were included in the TTH group, sex-specific effects and group-by-sex interactions could not be robustly examined. Therefore, the reported between-group differences, cut-off values, and diagnostic accuracy metrics should be interpreted with caution. Future studies should include larger, sex-balanced samples and prospectively stratify participants by clinically relevant subgroups to improve the precision and generalizability of discriminatory estimates. Finally, we focused on site-specific ROC analyses to identify anatomically informative PPT locations. Future studies should evaluate multivariable classification models combining PPT measurements from the most informative sites (e.g., temporalis and distal lower-limb locations), ideally in larger and sex-balanced cohorts with internal and external validation.

## 5. Conclusions

This study examined differences between individuals with migraine and TTH in both neural and muscular tissue sensitivity. Overall, participants with migraine showed lower PPTs than those with TTH across most evaluated sites, with the clearest differences observed at the temporalis muscles and, to a lesser extent, at distal lower-limb sites (particularly the tibialis anterior muscles and the left posterior tibial nerve). ROC and PR analyses suggest that these locations may have value as complementary diagnostic markers, with the temporalis region showing the most consistent discriminatory performance.

Importantly, a women-only sensitivity analysis yielded a broadly similar discriminatory pattern and, in some sites (particularly the temporalis muscles), slightly improved ROC-based metrics, supporting the consistency of the main signal while also indicating that sex composition may influence the magnitude of discrimination estimates and cut-off performance. However, the overall discriminative ability of PPTs was modest across most sites, and likelihood ratios indicate limited confirmatory power when used in isolation. Therefore, PPT assessment should be interpreted as an adjunct to clinical evaluation rather than as a stand-alone diagnostic tool. However, these conclusions need to be supported by further studies in larger and sex-balanced samples are needed to refine and validate more accurate discriminatory models.

## Figures and Tables

**Table 1:** PPT differences (adjusted for sex and age) observed between subjects with TTH and migraine.

Variables	TTH (*n* = 31)	Migraine (*n* = 33)	Difference (95% CI)	Adjusted Mean Difference (95% CI)	Adjusted *p* Value	Partial η*p*^2^
Left Temporalis muscle	2.08 ± 0.84	1.50 ± 0.57	0.58 (0.22; 0.94)	0.49 (0.10; 0.89)	0.015	0.094
Left C5/C6 zygapophyseal joint	1.73 ± 0.64	1.67 ± 0.51	0.06 (−0.22; 0.35)	0.03 (−0.3; 0.36)	0.847	0.001
Left 2nd and 3rd interdigital hand space	2.31 ± 1.08	1.96 ± 0.66	0.34 (−0.09; 0.79)	0.08 (−0.37; 0.53)	0.728	0.002
Left Tibialis anterior muscle	3.58 ± 1.85	2.63 ± 1.18	0.94 (0.17; 1.71)	0.90 (0.03; 1.78)	0.044	0.065
Left Arnold nerve	2.18 ± 0.95	1.85 ± 0.55	0.32 (−0.05; 0.71)	0.09 (−0.35; 0.52)	0.692	0.001
Left Radial nerve	1.89 ± 0.89	1.69 ± 0.48	0.19 (−0.15; 0.55)	0.03 (−0.35; 0.42)	0.863	0.000
Left Ulnar nerve	2.50 ± 1.06	2.17 ± 0.88	0.32 (−0.16; 0.81)	0.13 (−0.43; 0.69)	0.640	0.004
Left Median Nerve	1.61 ± 0.55	1.61 ± 0.53	0.00 (−0.27; 0.26)	0.11 (−0.19; 0.40)	0.466	0.009
Left Tibialis posterior nerve	2.64 ± 1.06	2.12 ± 0.60	0.52 (0.09; 0.95)	0.48 (−0.02; 0.98)	0.060	0.058
Left Fibular nerve	2.34 ± 0.96	2.20 ± 0.73	0.14 (−0.28; 0.56)	0.01 (−0.47; 0.48)	0.980	0.000
Right Temporalis muscle	2.16 ± 0.73	1.65 ± 0.68	0.51 (0.15; 0.86)	0.53 (0.13; 0.93)	0.011	0.105
Right C5/C6 zygapophyseal joint	1.77 ± 0.62	1.61 ± 0.50	0.15 (−0.12; 0.43)	0.03 (−0.28; 0.34)	0.853	0.001
Right 2nd and 3rd interdigital hand space	2.52 ± 1.15	1.93 ± 0.65	0.59 (0.12; 1.05)	0.37 (−0.13; 0.87)	0.139	0.036
Right Tibialis anterior muscle	3.61 ± 1.60	2.82 ± 1.07	0.79 (0.11; 1.47)	0.72 (−0.03; 1.47)	0.061	0.058
Right Arnold nerve	2.23 ± 0.87	1.80 ± 0.52	0.42 (0.07; 0.78)	0.18 (−0.21; 0.57)	0.367	0.014
Right Radial nerve	2.04 ± 1.01	1.72 ± 0.54	0.31 (−0.08; 0.72)	0.10 (−0.32; 0.53)	0.626	0.004
Right Ulnar nerve	2.73 ± 1.27	2.28 ± 0.89	0.45 (−0.09; 1.00)	0.13 (−0.47; 0.72)	0.671	0.003
Right Median Nerve	1.82 ± 0.76	1.74 ± 0.51	0.08 (−0.24; 0.40)	0.13 (−0.20; 0.47)	0.436	0.010
Right Tibialis posterior nerve	2.59 ± 0.97	2.15 ± 0.59	0.43 (0.03; 0.83)	0.31 (−0.15; 0.76)	0.183	0.029
Right Fibular nerve	2.56 ± 1.09	2.31 ± 0.71	0.24 (−0.21; 0.71)	0.11 (−0.42; 0.65)	0.669	0.003

**Table 2:** Discriminant capacity of PPTs at different locations and sides to differentiate patients with migraine from TTH.

Variables	ROC Value	95% CI	Cut-Off Point	Significance	Sensitivity	1-Specificity	Youden Index	Positive LR	Negative LR
Left Temporalis muscle	0.733	0.609–0.856	1.65	0.000	0.677	0.303	0.374	2.234	0.463
Left C5/C6 zygapophyseal joint	0.496	0.352–0.639	2.40	0.952	0.129	0.030	0.099	4.300	0.898
Left 2nd and 3rd interdigital hand space	0.580	0.436–0.725	2.50	0.277	0.452	0.152	0.300	2.974	0.646
Left Tibialis anterior muscle	0.663	0.528–0.799	2.45	0.018	0.710	0.333	0.376	2.132	0.435
Left Arnold nerve	0.581	0.437–0.725	2.45	0.272	0.323	0.091	0.232	3.549	0.745
Left Radial nerve	0.501	0.354–0.648	2.90	0.990	0.194	0.000	0.194	N/A	0.806
Left Ulnar nerve	0.586	0.445–0.727	2.05	0.231	0.645	0.455	0.191	1.418	0.651
Left Median Nerve	0.473	0.329–0.618	1.90	0.716	0.290	0.212	0.078	1.368	0.901
Left Tibialis posterior nerve	0.641	0.503–0.779	2.55	0.045	0.548	0.242	0.306	2.264	0.596
Left Fibular nerve	0.533	0.389–0.677	3.25	0.655	0.194	0.061	0.133	3.180	0.858
Right Temporalis muscle	0.707	0.581–0.833	1.95	0.001	0.484	0.182	0.302	2.659	0.631
Right C5/C6 zygapophyseal joint	0.556	0.414–0.697	1.05	0.440	0.903	0.788	0.115	1.146	0.458
Right 2nd and 3rd interdigital hand space	0.663	0.524–0.802	2.05	0.022	0.677	0.303	0.374	2.234	0.463
Right Tibialis anterior muscle	0.665	0.531–0.799	2.45	0.016	0.806	0.485	0.322	1.662	0.377
Right Arnold nerve	0.636	0.498–0.775	2.45	0.054	0.419	0.091	0.328	4.604	0.639
Right Radial nerve	0.557	0.412–0.702	2.40	0.440	0.323	0.182	0.141	1.775	0.828
Right Ulnar nerve	0.595	0.453–0.738	2.55	0.190	0.548	0.303	0.245	1.809	0.648
Right Median Nerve	0.489	0.343–0.634	2.35	0.880	0.226	0.061	0.165	3.705	0.824
Right Tibialis posterior nerve	0.624	0.484–0.764	2.60	0.083	0.484	0.182	0.302	2.659	0.631
Right Fibular nerve	0.546	0.403–0.690	2.65	0.527	0.484	0.333	0.151	1.453	0.774

**Table 3:** Discriminant capacity of PPTs analyses restricted to women.

Variables	ROC Value	95% CI	Cut-Off Point	Significance	Sensitivity	1-Specificity	Youden Index	Positive LR	Negative LR
Left Temporalis muscle	0.779	0.655–0.903	1.65	0.000	0.677	0.174	0.504	3.891	0.391
Left C5/C6 zygapophyseal joint	0.526	0.370–0.682	1.65	0.744	0.516	0.391	0.125	1.320	0.795
Left 2nd and 3rd interdigital hand space	0.590	0.437–0.744	2.45	0.247	0.452	0.130	0.321	3.477	0.630
Left Tibialis anterior muscle	0.703	0.561–0.845	2.45	0.005	0.710	0.261	0.449	2.720	0.392
Left Arnold nerve	0.532	0.367–0.687	2.45	0.683	0.323	0.130	0.192	2.485	0.778
Left Radial nerve	0.525	0.369–0.680	2.90	0.757	0.194	0.000	0.194	N/A	0.806
Left Ulnar nerve	0.582	0.428–0.736	2.45	0.295	0.516	0.348	0.168	1.483	0.742
Left Median Nerve	0.494	0.335–0.652	1.75	0.938	0.355	0.261	0.094	1.360	0.873
Left Tibialis posterior nerve	0.658	0.513–0.803	2.55	0.033	0.548	0.261	0.288	2.100	0.612
Left Fibular nerve	0.547	0.392–0.701	2.45	0.551	0.419	0.261	0.158	1.605	0.786
Right Temporalis muscle	0.752	0.625–0.879	1.95	0.000	0.484	0.130	0.353	3.723	0.593
Right C5/C6 zygapophyseal joint	0.566	0.408–0.723	1.15	0.412	0.871	0.739	0.132	1.179	0.494
Right 2nd and 3rd interdigital hand space	0.682	0.534–0.830	2.05	0.016	0.677	0.217	0.460	3.120	0.413
Right Tibialis anterior muscle	0.710	0.571–0.849	2.55	0.003	0.677	0.391	0.286	1.731	0.530
Right Arnold nerve	0.605	0.455–0.756	2.45	0.171	0.419	0.130	0.289	3.223	0.668
Right Radial nerve	0.572	0.418–0.725	1.85	0.362	0.484	0.261	0.223	1.854	0.698
Right Ulnar nerve	0.590	0.436–0.743	2.55	0.252	0.548	0.261	0.288	2.100	0.612
Right Median Nerve	0.475	0.318–0.631	2.35	0.752	0.226	0.043	0.182	5.256	0.809
Right Tibialis posterior nerve	0.628	0.479–0.777	2.60	0.092	0.484	0.174	0.310	2.782	0.625
Right Fibular nerve	0.542	0.387–0.697	2.75	0.595	0.452	0.304	0.147	1.487	0.787

## Data Availability

The original contributions presented in this study are included in the article. Further inquiries can be directed to the corresponding authors.
